# A Unique Case of an Arrow-Related Penetrating Spinal Cord Injury in Kenya and a Comprehensive Literature Review

**DOI:** 10.1055/a-2798-9778

**Published:** 2026-02-16

**Authors:** Filippos S. Chelmis, Fani C. Papacharalampous, Iliana N. Sorotou, Paraskevas Pakataridis, Hugh Williams, Josephat Mburu, Emmanuel Wekesa, Alexandru Budu

**Affiliations:** 1 Faculty of Medicine, Sofia University “St. Kliment Ohridski”, Sofia, Bulgaria; 2 Medical University Sofia, Faculty of Medicine, Bulgaria; 3 University Hospital “Lozenetz,” Sofia University “St. Kliment Ohridski,” Sofia, Bulgaria; 4 Department of Neurosurgery, Tenwek Hospital, Tenwek, Kenya; 5 Department of Neurosurgery, Queen Elizabeth Hospital, Birmingham, United Kingdom

**Keywords:** arrow injury, non-missile penetrating spinal cord injury, cervical spine, surgical management

## Abstract

**Background:**

Penetrating spinal cord injuries from arrows are rare. Arrowhead extraction can be challenging due to proximal critical neurovascular structures and tip variation. Our study highlights the most appropriate management plan based on our experience and current literature.

**Methods:**

Literature search on PubMed and Google Scholar was performed. This review examines optimal surgical management strategies, mean arterial pressure (MAP) evaluation, antibiotic protocols, and recovery timelines. Additionally, we investigate spinal cord decompression, focusing on its potential to reduce edema and accelerate recovery. Our study includes one case of a 32-year-old cervical penetrating spine injury caused by an arrow.

**Results:**

Literature recommends maintaining an MAP of 85 to 90 mm Hg for 7 days following blunt spinal cord injury. Evidence for penetrating injuries is limited and suggests no improvement with MAP augmentation. Prophylactic broad-spectrum antibiotics, for 48 hours, appear effective in preventing infection and early surgical intervention. Our patient had an incomplete spinal cord injury with preserved motor function in the right (dominant) hand and decreased motor function (3/5) in the left C8–T1 level. Careful planning must consider the shape of the arrow and the anatomy. Removal of the arrow tip requires proximal control, with additional consideration for dural repair.

**Conclusion:**

This case underscores the rarity and challenges of treating arrow-induced spinal cord injuries, particularly in resource-limited settings. Unlike blunt trauma, insufficient evidence supports elevated MAP or decompression for penetrating injuries. The primary management goal remains the safe, timely removal of the arrow and prophylactic antibiotics. Further research is needed to develop a standardized management protocol.

## Introduction

Non-missile penetrating spinal cord injuries (NMPSIs), especially those caused by arrows, are very uncommon and remain poorly reported in the literature. 1 2 This lack of documentation is notable, as the physical characteristics of arrows, including barbed tips and long shafts, complicate surgical removal and create specific risks for severe neurovascular compromise and infection. 3 4 Consequently, standardized management protocols are lacking. While early surgical decompression is recommended for blunt trauma, its efficacy in arrow injuries is less clear. 5 Additionally, strategies such as maintaining mean arterial pressure (MAP) lack strong proof for application in penetrating scenarios. 6 Infection risks further emphasize the need for specific antibiotic strategies. 7 In this article, we present a comprehensive literature review and a CAse REport guidelines (CARE)-compliant case report of a cervical arrow injury managed at Tenwek Hospital, Tenwek, Kenya. By reviewing this case alongside current literature, we aim to underscore the distinctive challenges of arrow-related penetrating spinal cord injuries and contribute to the development of evidence-based management protocols.

## Case Presentation

**Fig. 1:**
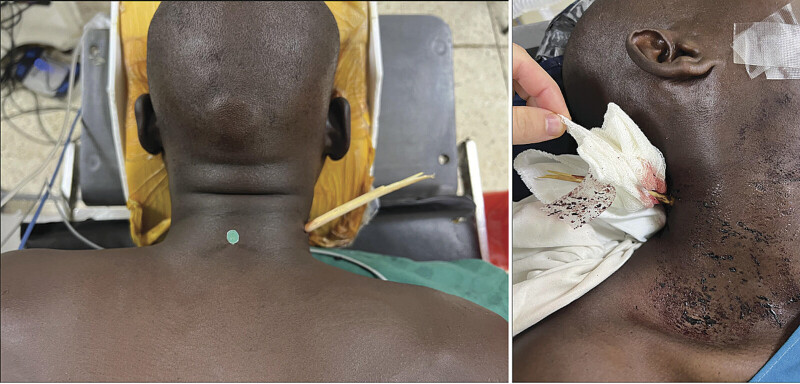
Transcervical penetrating injury from an arrow. Combined posterior and lateral views illustrate an entry point in the lower posterolateral cervical region.

**Fig. 2:**
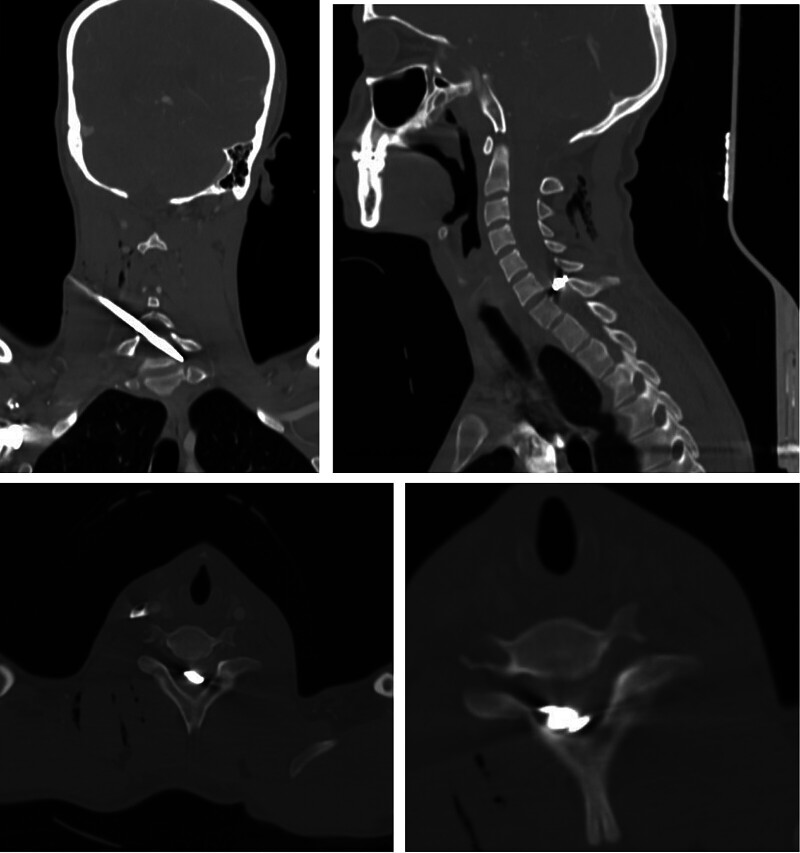
CT imaging demonstrating the trajectomy of a transcervical penetrating arrow injury with a retained metal tip/arrowhead. Axial, coronal, and sagittal CT cuts demonstrating a transcervical penetrating arrow injury with penetration of the spinal canal and cord section at C6–C7, accompanied by surrounding soft tissue emphysema.

A 32-year-old male from the Transmara region sustained a penetrating arrow injury to the posterior cervicothoracic junction during an ambush ( Fig. 1 ). He presented hemodynamically stable with an incomplete spinal cord injury. Examination revealed preserved motor function in the right upper limb, reduced strength (3/5) in left C8–T1 myotomes, and paralysis below the injury level. Intact anal tone and sacral sensation supported a diagnosis of American Spinal Injury Association (ASIA) B spinal cord injury at C7. High-resolution CT confirmed spinal canal penetration and cord transection ( Fig. 2 ). Given the limited ICU resources, urgent CT angiography was utilized to plan extraction. Surgical management involved cutting the external shaft and utilizing a posterior approach for proximal control. The embedded arrow tip was disconnected from the shaft, and the barbs were drilled and bent to minimize tissue trauma during removal. The dural defect was repaired with 5/0 Prolene and an autologous muscle-fascia patch. Postoperatively, respiratory depression was managed via CPAP to avoid invasive ventilation, and MAP was maintained at 75 to 80 mm Hg. The patient remained neurologically stable without worsening deficits. Clinical surveillance confirmed no CSF leak, infection, or sepsis. Physiotherapy was initiated, and the patient recovered without adverse reactions to the antibiotic or analgesic regimen.

## Discussion

### Non-Missile Penetrating Spinal Injury

NMPSIs present unique clinical challenges due to unclear management guidelines and variable mechanisms. 1 While rare in North America (0.8% of spinal cord injuries), NMPSIs account for up to 25% of cases in African regions due to sociopolitical factors. 8 These injuries predominantly result from violent assaults, affecting young males, and most frequently involve the thoracic (63%) and cervical (30%) spines. 1 6 8 9 10 We present a rare case of cervical arrow injury managed in a resource-limited setting.

### Neurological Sequelae and ASIA Score

Clinical presentation varies from asymptomatic dural tears to complete transection. Prognosis is generally favorable (50–60% recovery), as low-velocity weapons cause less cavitation than high-velocity projectiles. 6 11 Neurological deficits result from direct disruption, vascular compromise, or secondary compression via hematoma or edema. Delayed complications often stem from retained foreign bodies, making objective assessment via the ASIA impairment scale essential for detecting decline and guiding surgical decision-making. 1

### Radiographic/Neuroimaging Studies

Plain radiographs and CT scans are recommended to localize foreign bodies and assess osseous structures. 12 13 While X-rays are accessible in resource-limited settings, CT offers superior 3D visualization of the trajectory. 11 Postoperative MRI is valuable for assessing soft tissue, but is often unavailable. In cervical NMPSI, angiography is strongly recommended to evaluate vertebral or carotid artery integrity. 6

### Arrow-Related Spinal Injury and Management

Management adheres to advanced trauma life support (ATLS) principles; blind removal is contraindicated as it may precipitate hemorrhage or neurological deficit. 14 15 A review of eight arrow-related SCIs (mean age 25.4 years) showed a predilection for the cervical spine (75%; Table 1 ).

**Table 1:** Summary of Reported Arrow-Related Non-Missile Penetrating Spinal Cord Injuries

Study	Age (years)	Gender	Level	Injury signs (motor disturbances, etc)	ASIA score	Surgery (spine, decompression, etc)	MAP	Antibiotics	Outcome (recovery, death)
Carlstrom et al 4	8	M	T6	Progressively mobilized and did not develop signs of CSF leak or fistula	D	The arrow was surgically removed without complications through an anterior-only approach	Yes, pressors were used mean arterial pressure goal of greater than 65 mm Hg	Prophylactic broad-spectrum antibiotics with vancomycin, ceftriaxone, and metronidazole	Remarkable neurologic recovery
Ismail et al 3	19	M	C6	Alert, oriented, not pale, not cyanosed with stable vitals	N/A	Emergent neck exploration and arrow removal under general anesthesia	N/A	Postoperatively, was placed on intravenous ceftriaxone 2 g once daily	No complications and has resumed his normal daily life routine
Skadorwa and Ciszek 7	11	M	C4–C5	Did not present any neurological deficit nor paresthesia	N/A	Yes, a bilateral C4–C5 hemilaminectomy was performed to verify the spinal cord injury and facilitate dural suturing, which can be considered a form of decompression	N/A	Yes, IV (ceftriaxone, amikacin, and metronidazole) for 2 weeks postsurgery	Yes, discharged after 2 weeks in complete health, with no neurological deficits; follow-up after 6 months confirmed stability
Gupta et al 16	12	F	C7	Conscious, had no difficulty in breathing or deglutition, and had no weakness in her limbs after the impact	N/A	The surgeon performed exploration of the posterior triangle of the neck. They could release the arrow from subcutaneous tissue and cut the arrow close to the skin, which enabled the patient to adduct the arm. They ruled out injury to aerodigestive tracts and major neck vessels and found that arrow was stuck in the vertebral body	N/A	N/A	Yes, the patient has completed 6 months postsurgery, is independent in daily activities, and enjoys good neck movement
Kolena et al	17–20	F	L2 vertebra	N/A	N/A	N/A	N/A	N/A	Death
Kovari 17	57	M	C2 vertebra	Somnolent and was able to communicate only by moving his limbs, eyes, and eyelids. He was able to move all his extremities against gravity, and major palsy was not detected	N/A	Yes, a C2 laminectomy was performed to access the spinal canal and remove the arrow shaft, aiding in decompression of the cord	N/A	Yes, prophylactic intravenous antibiotics were administered, initially amoxicillin/clavulanic acid, later adjusted for *Escherichia coli* meningitis.	Yes, after 4 months, the patient showed improvement in fine motor skills, could walk unsupported, and had no major palsy
Nepal et al 19	32	M	C7 vertebra	On examination, the patient was in recumbent position on bed supported by attendants, slight respiratory difficulty but there was no associated hemoptysis, dysphasia, or hoarseness. No surgical emphysema was noted around the neck. There was no neurological deficit	N/A	The patient was taken to the emergency operating theater. He was intubated in right lateral position as the arrow was protruding out through the left side of neck posteriorly. The neck was explored by giving entry to exit lazy “S”-shaped incision	N/A	IV antibiotics	Follow-up for the last 6 months was normal
Jamtsho and Pradhan 20	46	M	C2–C3	Stable with no loss of consciousness or bleeding	N/A	Under general anesthesia, the entry site was widened, and meticulous tissue dissection was performed to safely remove the arrow	N/A	N/A	The patient is reported to be doing well after a period of 2 weeks

Abbreviation: MAP, mean arterial pressure.

Analysis of eight reported cases revealed a mean age of 25.4 years (range: 8–57). Injuries predominantly involved the cervical spine (75%), with single instances in the thoracic and lumbar regions. Formal ASIA scoring was rare (one ASIA D), and presentations ranged from stable to respiratory compromise. Management included antibiotics (62.5%) and pressors (12.5%), while surgical intervention typically involved arrow removal, and laminectomy was performed in 87.5% of patients. Outcomes were largely favorable (87.5% recovery), with one fatality (12.5%). The treatment algorithm relies on removing retained foreign bodies to prevent infection, CSF leak, and neurological deficit. Intervention typically entails a laminectomy, hemostasis, and dural repair. 14 This approach aligns with established principles from high-volume African centers and our literature review, 16 validating the high frequency of surgical intervention (87.5%) for retained arrow injuries. This necessity was reflected in our case, where emergent neurosurgical removal formed the basis of treatment. While certain less complex penetrating injuries might allow for non-operative consideration, the presence of a retained, structurally complex foreign body like an arrow generally requires operative removal, aligning this case and the reviewed cohort with optimal management methods for such scenarios. 6 7 8 14 17 The principle supporting early surgical intervention to mitigate complications and potentially reduce infections, as highlighted in the literature, was clearly adopted through the emergent nature of the surgery performed, likely contributing significantly to the positive outcome. Although precise intervention timing was not a focus of the arrow cohort summary, their predominantly favorable recovery rate (87.5%) suggests that timely surgery was likely a common feature. Furthermore, the surgical technique employed for object removal adhered closely to literature recommendations: Meticulous stabilization of the foreign body during the careful removal of adjacent lamina, frequently involving laminectomy, to facilitate atraumatic extraction. Our case report elucidates this further by detailing specific technical adaptations necessitated by the barbed arrowhead, requiring careful drilling around the embedded barbs and strategic manipulation to minimize further neural insult during retraction. This approach addresses the unique biomechanical challenge posed by such barbs. While data suggest a relatively small hemorrhage risk (∼5%) associated with the arrowhead removal, the complex technique detailed for the arrow underscores the heightened caution warranted due to the increased potential for neural or vascular compromise with barbed tips. It is crucial to highlight that the management of our patient was conducted within a resource-limited setting, which introduced substantial intraoperative and postoperative difficulties. These limitations undeniably increased the inherent complexity and potential risks of the operation, requiring exceptional surgical precision and meticulous planning to manage the extraction of barbed arrow tips from the spine without causing further injury. Regarding dural management following such injuries, there exists a huge debate among surgeons concerning the absolute necessity of primary dural closure. 4 Some case reports recommend meticulous repair, mentioning potential risks like pneumocephalus, persistent CSF leakage, subsequent infections, or even neurological decline associated with an unclosed durotomy. On the other hand, other experiences, including instances where careful foreign body removal was performed without direct dural repair, have yielded good outcomes without these feared sequelae, suggesting that larger studies may not consistently prove the supposed high risks of excluding formal closure, potentially influenced by factors like the specific location and nature of the dural defect. In our presented case, however, the decision was made to proceed with a primary dural repair utilizing an autologous fascia and muscle patch graft. This approach aligns with the principle of restoring anatomical integrity where possible and directly addresses the potential for CSF leak. This strategy yielded an excellent outcome, with no clinical signs of CSF leak or complications postoperatively. Hemodynamic management in NMPSIs remains debated. Current guidelines recommending MAP goals of 85 to 90 mm Hg for non-penetrating SCI exclude penetrating injuries, 1 and data from gunshot wounds suggest minimal benefit from such aggressive targets. Consequently, clinical practice varies; while one reported arrow injury maintained MAP >65 mm Hg, our patient was managed at 75 to 80 mm Hg. 4 These discrepancies highlight the urgent need for NMPSI-specific hemodynamic protocols. Similarly, the antibiotic strategy lacks clear guidelines, though the risk associated with retained fragments strongly supports prophylaxis. While infection rates vary from 2% to 4%, generally to 18% in stab wounds without prophylaxis, preventative treatment is widely advocated. 1 14 18 Consistent with our review of arrow NMPSI cases, we employed broad-spectrum antibiotics. 3 4 7 17 19 However, significant regimen variability persists, underscoring the need for definitive evidence regarding selection and duration.

## Conclusion

This case highlights the extreme rarity and complexity of arrow-induced NMPSIs, especially in low-resource settings. There is limited literature and no standardized protocols for managing such injuries, particularly involving the cervical spine, leaving clinicians with minimal guidance. Recommendations for MAP control, surgical management, or antibiotic use remain unclear in penetrating cases. Financial and infrastructural barriers further complicate both surgical intervention and postoperative care. This case and comprehensive literature review emphasize the need for more research, evidence-based guidelines, and international efforts to support neurosurgical care in underresourced regions.
